# Correction: Competition and cooperation: The plasticity of bacterial interactions across environments

**DOI:** 10.1371/journal.pcbi.1013809

**Published:** 2026-01-15

**Authors:** Josephine Solowiej-Wedderburn, Jennifer T. Pentz, Ludvig Lizana, Bjoern O. Schroeder, Peter A. Lind, Eric Libby

## Notice of Republication

This article was republished on November 12, 2025, to correct Fig 1. Please see corrected figure.

**Fig 1:**
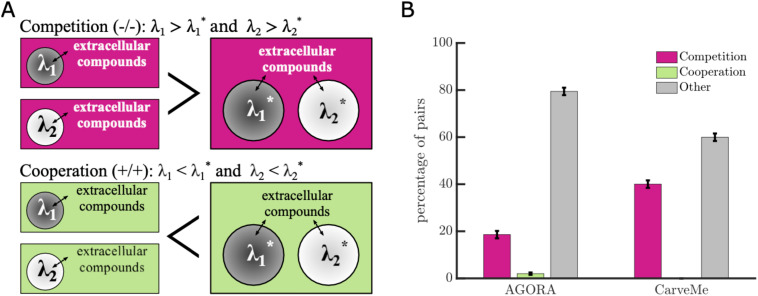


## Supporting information

S1 File Originally published, uncorrected article. (PDF)

S2 File Republished, corrected article. (PDF)
