# Supplementary material for: Competition and cooperation: The plasticity of bacterial interactions across environments
Source: PLoS Comput Biol. 2025 Jul 24;21(7):e1013213. doi: 10.1371/journal.pcbi.1013213 (PMC12289095; doi:10.1371/journal.pcbi.1013213)
Supplement: S1 Table — The 10 most common essential compounds for pairs of bacteria in AGORA (a) and CarveMe (b). (PDF) [file pcbi.1013213.s023.pdf]

Table S1. Common essential compounds. The 10 most common essential compounds for pairs of bacteria in AGORA (a) and CarveMe (b).

(a)

Compound	% pairs
zinc	100%
manganese	100%
magnesium	100%
potassium	100%
copper (2+)	100%
cobalt (2+)	100%
chloride	100%
calcium (2+)	100%
sulfate	97.2%
thiamin	89.4%

(b)

Compound	% pairs
zinc	100%
magnesium	100%
potassium	100%
cobalt (2+)	100%
chloride	100%
calcium (2+)	100%
manganese	99.9%
oxygen	89.6%
sulfate	82.0%
citrate	52.1%
