# Supplementary material for: Competition and cooperation: The plasticity of bacterial interactions across environments
Source: PLoS Comput Biol. 2025 Jul 24;21(7):e1013213. doi: 10.1371/journal.pcbi.1013213 (PMC12289095; doi:10.1371/journal.pcbi.1013213)
Supplement: S2 Table — Top ten compounds responsible for transitions a) competition to facultative cooperation; b) facultative cooperation to competition; c) competition to obligate; d) facultative cooperation to obligate in AGORA pairs considered in S9 Fig. (PDF) [file pcbi.1013213.s024.pdf]

Table S2. Compounds which cause transitions in AGORA. Top ten compounds responsible for transitions a) competition to facultative cooperation; b) facultative cooperation to competition; c) competition to obligate; d) facultative cooperation to obligate in AGORA pairs considered in Fig S9.

(a) Competition to facultative cooperation

Compound	% switches
L-threonine	8%
proton	6%
water	5%
glycylleucine	4%
oxygen	4%
L-glutamate(1-)	3%
L-aspartate(1-)	3%
L-asparagine	3%
acetaldehyde	3%
L-argininium(1+)	3%

(b) Facultative cooperation to competition

Compound	% switches
oxygen	12%
cytidine	5%
nitrate	4%
2-oxobutanoate	4%
glycylleucine	3%
L-threonine	3%
L-argininium(1+)	3%
Adenosine	3%
nitrite	2%
L-alanyl-L-threonine	2%

(c) Competition to obligate

Compound	% switches
nicotinate	10%
hydrogen phosphate	7%
L-valine	6%
L-tryptophan	4%
L-isoleucine	4%
L-argininium(1+)	4%
L-asparagine	4%
octadecanoate	3%
riboflavin	3%
L-histidine	3%

(d) Facultative cooperation to obligate

Compound	% switches
nicotinate	9%
hydrogen phosphate	7%
L-valine	6%
L-tryptophan	4%
L-isoleucine	4%
L-argininium(1+)	4%
L-asparagine	3%
L-lysinium(1+)	3%
riboflavin	3%
octadecanoate	3%
