# Supplementary material for: Competition and cooperation: The plasticity of bacterial interactions across environments
Source: PLoS Comput Biol. 2025 Jul 24;21(7):e1013213. doi: 10.1371/journal.pcbi.1013213 (PMC12289095; doi:10.1371/journal.pcbi.1013213)
Supplement: S3 Table — Top ten compounds responsible for transitions a) competition to facultative cooperation; b) facultative cooperation to competition; c) competition to obligate; d) facultative cooperation to obligate in CarveMe pairs considered in S10 Fig. (PDF) [file pcbi.1013213.s025.pdf]

Table S3. Compounds which cause transitions in CarveMe. Top ten compounds responsible for transitions a) competition to facultative cooperation; b) facultative cooperation to competition; c) competition to obligate; d) facultative cooperation to obligate in CarveMe pairs considered in Fig S10.

(a) Competition to facultative cooperation

Compound	% switches
L-arginine	5%
fumarate	5%
water	3%
hydrogen sulfide	3%
nitrite	3%
glycerol 3-phosphate	2%
L-glutamate	2%
acetaldehyde	2%
L-tartrate	2%
nitrate	2%

(b) Facultative cooperation to competition

Compound	% switches
nitrate	7%
4-aminobutanoate	5%
2-oxoglutarate	5%
L-glutamate	4%
D-serine	4%
putrescine	3%
nitrite	3%
reduced glutathione	2%
L-glutamine	2%
nitric oxide	2%

(c) Competition to obligate

Compound	% switches
copper 2+	11%
iron 3+	11%
iron 2+	11%
benzoate	7%
L-arginine	4%
hydrogen phosphate	3%
L-asparagine	3%
2-phosphoglycolate	3%
salmochelin-S4-Fe-III	3%
oxygen	2%

(d) Facultative cooperation to obligate

Compound	% switches
copper 2+	10%
iron 2+	10%
iron 3+	10%
benzoate	7%
hydrogen phosphate	4%
L-arginine	4%
2-phosphoglycolate	3%
L-lysine	3%
L-asparagine	2%
oxygen	2%
