# Supplementary material for: Competition and cooperation: The plasticity of bacterial interactions across environments
Source: PLoS Comput Biol. 2025 Jul 24;21(7):e1013213. doi: 10.1371/journal.pcbi.1013213 (PMC12289095; doi:10.1371/journal.pcbi.1013213)
Supplement: S1 Appendix — (PDF) [file pcbi.1013213.s026.pdf]

Appendix S1. Mechanistic interpretation of transitions in case study Fig 5.

In the main text we found that the difference between competition, facultative cooperation or an obligate interaction between the same pair of bacteria could come down to a single compound. Having isolated compounds that cause interaction switches when removed from an environment, we might want to investigate the mechanisms underlying the switches. We could approach this by first identifying the metabolic reactions in both microbes that involve one of these compounds. Then running the flux balance analysis would give a set of reactions that are being used by the microbes before and after the compound is removed (however, it is important to note that while the maximized growth rate has a unique solution, the reaction fluxes to achieve this are not unique). Ideally, these analyses would identify consistent links between particular compounds, the pathways they are used in, and consequence on microbial interactions. For example, if two microbes have optimal pathways that both rely on the same compound, they would compete for it; on the removal of that compound the microbes would be forced to choose different pathways which might open up opportunities for cooperation if the alternative pathways produce favorable by-products. In the case of a switch to obligate interactions, one possible scenario is that the given compound is critical for the growth of one of the microbes which is unable to synthesize it itself (is auxotrophic). Hence, if the compound is removed the microbe becomes obligate on its partner (who in this scenario is able to synthesize and export the compound).

We investigated the case studies in Fig. 5 in search of such mechanisms. We focused on switches to obligate interactions as we thought that these would present the cleanest story. Among these examples we could not identify any immediate auxotrophies as outlined above. However, we still found many examples of obligate interactions in our case studies. For example, of the 70 cases of switches to obligate interactions between *Prevotella copri DSM 18205* and *Ruminococcus gnavus AGR2154*, 30 were caused by the removal of L-glutamine and 13 by the removal of L-glutamate(1-)—two amino acids which are present in the biomass reactions of both bacteria indicating that they are necessary for growth. While the *Ruminococcus* strain can import/export both L-glutamine and L-glutamate, the *Prevotella* strain can only import/export L-glutamate. Both can convert L-glutamine to L-glutamate and vice versa. We found that in some environments lacking both L-glutamate and L-glutamine, *Prevotella* was still able to synthesize both amino acids from the other environmental compounds present; however, it was often the case that while *Ruminococcus* could make up for a lack of either L-glutamine or L-glutamate in the environment it could not grow without both. In such cases, when L-glutamine was removed, *Prevotella* which lacks a transport reaction for L-glutamine continued to be able to grow, whereas *Ruminococcus* could no longer grow by itself but became obligate on *Prevotella*.

Delving deeper into the mechanisms, we sought to identify which metabolic reactions both species used in the 30 cases when *Ruminococcus* became obligate on *Prevotella* when L-glutamine was removed. These switches all come from the same starting environment, which was lacking in L-glutamate, but with different sequences of compounds removed (Fig 5G). Here, we observed an average of 16.7 reactions (range 8–26, spanning 81 unique reactions) that *Prevotella* began to use after L-glutamine was removed from the environment, while *Ruminococcus* stopped using an average of 10.2 reactions (range 6–16, spanning 80 unique reactions) and started using an average of 11.1 reactions (range 5–19, spanning 85 unique reactions) after L-glutamine was removed. In some cases, it is clear to see how the obligate relation could be realized, for example, in 11/30 cases *Prevotella* began to export L-glutamate after L-glutamine was removed which could then directly be imported and used by *Ruminococcus*. However, even in these cases, providing a full explanation of all the internal metabolic reactions that start/stop being used with/without L-glutamine is less obvious. Indeed, to explain each reaction change may not be entirely meaningful since the flux balance analysis only outputs one possible set of reactions that could give the maximized growth output, whereas there may be many alternative metabolic reactions or pathways that give the same result. For some specific cases of lab-cultured strains metabolic models have been carefully curated to partition reactions into different pathways and incorporate regulatory mechanisms (see, e.g., (1–3)), but this has not been done on a broader scale. As metabolic models become refined, we can hope to achieve stronger predictions and better understandings of the complex interplay between resource availability, metabolic pathways and their effect on microbial interactions.

Our analysis further highlighted the environmental context of interaction switches. For example, we found 5 cases where the removal of deoxycytidine resulted in a switch competition/cooperation to an obligate interaction between *Prevotella Ruminococcus*, however, in 10 cases it could be removed with no effect (here, competitive interactions remained competitive). Furthermore, when deoxycytidine was removed from environments with *Prevotella* and *Bacteroides vulgatus ATCC 8482* there was no change in the majority of cases—31 competitive interactions remained competitive and 29 cooperative interactions remained cooperative—however, we also found 1 case where the removal of deoxycytidine could switch their interaction from facultative cooperation to competition and 15 cases where its removal switched the interaction to obligate. In the context of these case studies, environments only differ by a few compounds and yet we can see a variety of interaction shifts. Furthermore, in many cases, we observed changes in over 200 reactions of a single metabolism when a single compound was removed, hence making it difficult to isolate a single critical metabolic pathway responsible for an interaction switch. In the same way that we found a range of different metabolic mechanisms that could account for interaction switches between a specific pair, there was an even wider range of possible mechanisms between different

pairings.

1. Jeffrey D Orth, Tom M Conrad, Jessica Na, Joshua A Lerman, Hojung Nam, Adam M Feist, and Bernhard Ø Palsson. A comprehensive genome-scale reconstruction of escherichia coli metabolism—2011. *Molecular systems biology*, 7(1):535, 2011.
2. Anu Raghunathan, Jennifer Reed, Sookil Shin, Bernhard Palsson, and Simon Daefler. Constraint-based analysis of metabolic capacity of salmonella typhimurium during host-pathogen interaction. *BMC systems biology*, 3:1–16, 2009.
3. Niels Klitgord and Daniel Segrè. Environments that Induce Synthetic Microbial Ecosystems. *PLOS Computational Biology*, 6(11):e1001002, November 2010. ISSN 1553-7358. doi: 10.1371/journal.pcbi.1001002. Publisher: Public Library of Science.
