# Supplementary material for: Competition and cooperation: The plasticity of bacterial interactions across environments
Source: PLoS Comput Biol. 2025 Jul 24;21(7):e1013213. doi: 10.1371/journal.pcbi.1013213 (PMC12289095; doi:10.1371/journal.pcbi.1013213)
Supplement: Attachment — Submitted filename: Reviewer_Response.pdf [file pcbi.1013213.s027.pdf]

Competition and cooperation: The plasticity of bacterial interactions across environments - Response letter to reviewer comments

Overview

Response: We thank the editor and reviewers for reading and evaluating our paper. The comments were very helpful and by addressing them we think the manuscript is much improved. Here we address the comments of both the editor and reviewers point by point. Comments are in blue font and our response follows in black.

Editor

The manuscript lacks some clarifications and would benefit from addressing the issues. Some of the major issues are conceptual but others also relate to the methods used and their opacity. E.g. both the reviewers in their major point #2 address the issue of how to optimally address the environment similarity dissimilarity measure but lacks a metric.

Response: Thank you for highlighting these issues in clarity, it helped to organize our revisions. We have systematically gone through the manuscript making clarifications, especially in areas flagged by the reviewers. For example, we now include analyses of environmental similarity using the Jaccard distance (see Supp Fig. S7). We have made many other changes and described them in our responses to specific comments by the reviewers.

Making definitions clear and stating them upfront along with the assumptions also seems to be common point of issue for both the reviewers which I would tend to agree too with my reading of the manuscript.

Response: We have now restructured part of the Introduction so as to make it much clearer how we define cooperation and competition and the scope for our analyses, i.e. assuming selection is acting on growth. These changes provide the appropriate context for our work much earlier on in the paper, thereby avoiding confusion.

Lastly I would urge to make clear the statistical methods and perhaps deposit them along side the other code and data in a repository that supports doi registration. As Reviewer 2 mentions, Zenodo might be an option.

Response: We have now been through all the figures in the main text and made sure that they have clear and appropriate statistical analyses. For example, we have added Supp fig. S22 to show the statistical significance of the obligate switches that we observed in the environmental degradation analyses. The code for all the statistical tests has now been added to the github repository which we have also deposited on Zenodo as recommended by Reviewer 2 (https://doi.org/10.5281/zenodo.15075550).

Reviewer 1

The manuscript provides an innovative computational framework to explore the plasticity of microbial interactions across diverse environmental conditions using genome-scale metabolic models. Analyzing 10,000 bacterial pairs across thousands of simulated environments from AGORA and CarveMe databases, the authors reveal that resource depletion often drives interactions toward obligate cooperation. These findings are valuable for understanding microbial ecology and synthetic community design. However, the manuscript would greatly benefit from addressing several key methodological and conceptual gaps, particularly by expanding the perturbation analysis and integrating it with metrics of environmental similarity. Below, I outline the major and minor points requiring attention to strengthen the manuscript.

Response: Thank you for the positive response to the computational framework and the results of our analyses. We have made several revisions to address issues concerning the methodological and conceptual framework and discuss them in subsequent responses.

Major Points

1. Perturbation Analysis Framework
The current perturbation analysis focuses on the removal of single compounds to assess the robustness of microbial interactions. However, this approach does not differentiate between redundant and non-redundant compounds. Removing a redundant compound may have minimal ecological impact, whereas removing an essential, non-redundant compound could drastically alter interaction dynamics. Incorporating redundancy metrics—based on functional or structural similarity—would provide a more realistic and biologically meaningful framework for perturbation analysis.

Response: We completely agree that not all compounds are the same and their role in metabolism affects how they contribute to the pair-wise interaction. For the analyses in our paper we actually did draw a distinction between compounds based on their use, or redundancy, though it perhaps was not stated clearly or explicitly. We have now modified the text to make this clear, adding the sentence: "*To mitigate the effect of redundant compounds which would have minimal ecological impact, we identified the compounds being 'used' by at least one species on a pair-by-pair basis and reduced environments to contain only these compounds before performing our degradation analysis (see Fig. S1).*" when we describe the perturbation analysis in the Results. Where fig. S1 is an extra schematic in the supplement to clarify our measures of redundancy, i.e. defining essential vs substitutable and used vs redundant compounds.

Regarding the reviewer's comment, we also agree it could be useful to somehow incorporate functional or structural similarity information about compounds into our analyses of the effects of environmental perturbations on microbial interactions. However, it is not a straightforward task to determine the role of each individual compound in a metabolism and which pathways it is involved in. This information will vary both across microbes (depending on their specific metabolisms) and within a particular microbe (depending on the abundance of that compound), e.g. https://doi.org/10.1371/journal.pbio.3002482, https://doi.org/10.1038/s41559-021-01535-8, https://doi.org/10.1038/s41467-024-47594-w. Moreover, if two compounds are metabolically similar, it may be possible that only one can be processed by a particular microbe. Adding to the challenge, chemical similarity is not necessarily a proxy for metabolic similarity, e.g. two chemically-identical compounds with different chirality can have drastically different metabolic effects. For instance, in the context of the human gut, dietary fiber (or host mucins) commonly consist of chemically similar monosaccharide units that differ in the connectivity of their glycosidic bonds. These patterns of connectivity determine whether the compound can be utilized by a particular microbe or not. Hence, any redundancy metric will vary with the metabolism, and may also change depending on which metabolism it is paired with.

In our analyses we circumvented these challenges by determining redundancy on a pair by pair basis. When we used flux balance analysis to predict growth rates, we also obtained information concerning which compounds from the environment were used (i.e. acted on by some chemical reaction) or ignored (i.e. never used as a substrate or produced as a product of a reaction). These ignored compounds can be considered 'redundant' because removing them will not affect any other reaction. For our perturbation analyses we began by removing all such unused, 'redundant' compounds precisely because we knew they would have no effect and were in some sense mathematically trivial. While our approach to redundancy does not take into account any functional or structural information about compounds, it does use empirical information about their use in specific environmental/ecological contexts. Thus it ensures that every compound we removed in the perturbation analysis was a used (or non-redundant) compound with some metabolic use to at least one microbe in the specific environment.

> *Furthermore, this analysis could naturally integrate with the concept of environmental similarity (point 2 below). By characterizing environments based on molecular similarity or dissimilarity, the study could explore whether interactions are more resilient to changes in chemically similar environments versus divergent ones. This connection would allow the authors to investigate whether resilience is a function of environmental redundancy or diversity. Expanding the perturbation analysis in this manner would add significant depth and broaden the study's implications.*

Response: We agree that integrating a characterization of the environmental compounds would add depth to the study. Ideally, we would have a classification scheme that could distinguish between environments based on the types of compounds they contain. One challenge is that it is not obvious what characteristics of compounds should be used to build the classification scheme. For example, a metabolic pathway analyzer (such as https://www.metaboanalyst.ca/MetaboAnalyst/upload/PathUploadView.xhtml) could give an indication of which pathways the environmental compounds are involved in. However, this will strongly depend on the metabolisms of specific pairs and the abundances of compounds. Another option would be to classify compounds by their functional groups using, e.g. the KEGG database of compounds with biological roles (https://www.genome.jp/brite/br08001). But again, as different microbes have different metabolic pathways the role of compounds will vary across metabolisms. Thus, it would be extremely challenging to objectively classify environments as similar or dissimilar.

As a simple example, imagine that there are two species growing in an environment with a carbohydrate that can be used for growth. If both species have the metabolic pathways to import and process the carbohydrate then they may compete for it. If instead, neither species can fully use it but together they can, then they may cooperate. Finally, if one species can use it and the other cannot then the two species may have a neutral interaction. Regardless of how we characterize the compound we will fail to accurately distinguish between these environments. Therefore, even though we fully agree with the reviewer's suggestion, we found it impossible to solve this challenge (in general) with the present databases in an unbiased manner. Nonetheless, we performed an initial exploration using a motivated, but somewhat biased, compound characterization that we describe below.

To address the reviewer's comment, we chose to focus on the presence of the 20 proteinogenic amino acids in environments. Since amino acids have been found to play a key role in shaping ecological interactions (e.g. https://doi.org/10.1038/s41564-023-01511-x, https://doi.org/10.1039/C8NP00009C), we thought they would be a good candidate set of compounds. As a comparison case, we also identified two groups of simple sugars—aldoses and ketoses—which we expected to have a characteristically different influence on individual metabolisms and consequent interactions between pairs. We tested how frequently the removal of each of these compounds resulted in a switch from a competitive to a facultative cooperative or obligate environment, or from a facultative cooperative environment to competitive or obligate. In accordance with our previous analysis of the most common compounds to cause switches we found that the removal of amino acids from environments commonly caused switches to obligate interactions (in AGORA 16 amino acids were responsible for significantly many more switches from competitive to obligate environments than expected if each compound was equally likely to cause

a switch based on the averages from fig. 4 and 18 from cooperative to obligate; in CarveMe 15 amino acids caused significantly more switches to obligate interactions from both competitive and cooperative environments, *p*-value< 0.01). While the majority of amino acids were responsible for a significant number of switches to obligate interactions when removed, approximately half were also responsible for a significant number of switches between competitive and facultative cooperative interactions.

Conversely, the removal of a simple sugar from the environment is hardly ever responsible for a switch to an obligate interaction (and none are statistically significant). However, across both AGORA and CarveMe we do find that the removal of D-fructose is responsible for a statistically significant number of switches from cooperative to competitive environments (*p*-value< 0.01); in CarveMe D-glyceraldehyde and D-fructose are both responsible for significantly more switches to cooperative and competitive environments. All this data is now presented as additional supplementary figs. S13 and S14, and highlighted in the main text: *"In particular, several amino acids were found to cause all types of transitions for both collections, with most (15–18) responsible for a statistically significant proportion of switches to obligate interactions and 7–15 responsible for significant proportions of switches between competitive and facultative cooperative interactions (p-value< 0.01, fig. S13). (...) By contrast, the removal of simple sugars from the environment was less common to cause a change in the interaction between pairs of bacteria (fig. S14)."*

Taken together, these results indicate that different groups of environmental compounds can have characteristically different effects on the interactions between bacteria. Within these groups, individual compounds can also have different effects. As the removal of some compounds can cause significant numbers of transitions between both competitive and cooperative interactions, we are reminded that the combination of compounds and individual microbial pairings must also be taken into account. We believe that delving deeper into this would be a very interesting analysis. For a first step it would require a more thorough characterization of compounds than the two groups we identified. To be done properly, this characterization should also be integrated with the metabolism of each specific pair which we believe could present another paper in itself. Indeed, we are exploring this as part of a more general framework.

2. Environmental Similarity and Diversity
While the manuscript emphasizes the impact of environmental diversity (i.e., the number of compounds) on microbial interactions, it neglects the molecular composition of these environments. Chemically similar environments may yield comparable interaction dynamics, while divergent environments might drive distinct patterns. Introducing a metric for environmental "distance" (e.g., based on molecular functional groups or structural similarity) would provide new insights into how microbial interactions respond to environmental variability.

Response: Thank you for this suggestion! We think that incorporating a metric for the "distance" between environments would provide insightful understanding into the structure of environments that promote different types of interactions. In this light, we used the Jaccard distance to quantify the similarity of two environments based on the overlap of compounds between them. Two identical environments would have a Jaccard distance of 0, whereas completely distinct environments would have a Jaccard distance of 1. We found mean Jaccard distances of 0.5–0.82 between both environments of the same type (competitive or cooperative) and when comparing across both types of environment (competitive vs cooperative), thus indicating that we found a diverse spread of environments where bacteria can compete or cooperate with moderate overlap. While there was a statistically significant difference (ANOVA p-value < 0.001) in the distance between environments of the same type and comparing across both types of environment, this difference was small ($< 4\%$) indicating that it would be difficult to distinguish between competitive and cooperative environments with a simple similarity metric.

To determine whether it was easier to distinguish between competitive and cooperative environments for individual pairs, we selected a set of pairs where we found at least 5 of each type of environment. We then calculated the Jaccard distance between competitive environments and between cooperative environments, as well as between competitive and cooperative environments. On average, the Jaccard distance between environments for a particular pair was smaller than the distance between random

environmental pairings across different pairs. This finding shows that environments where a particular pair can grow are more similar than comparing across pairs. However, an ANOVA shows that there is no significant difference (all p-values $> 5\%$) in the Jaccard distance between the same type of environment and competitive vs cooperative. This suggests that it is not possible to distinguish between competitive and cooperative environments with a simple similarity metric on a pair-by-pair basis. Indeed, determining whether there is some way to distinguish signatures of competitive and cooperative environments taking a more holistic account of the combinations of compounds present is an on-going research question that we are continuing to investigate. These results are now included as a new paragraph in the main text. Here is an excerpt: "*We first applied this metric [Jaccard distance] within competitive and cooperative environments and found a diverse spread of environments for both (mean distance 0.58–0.82, Fig. S7A–D). We then calculated the Jaccard distance between competitive and cooperative environments and found similar distances (means 0.60–0.81), indicating that it is difficult to distinguish between competitive and cooperative environments with a simple similarity metric, even on a pair-by-pair basis (Fig. S7E–H).* " Where, fig. S7 is an additional supplementary figure of the Jaccard distance between competitive and cooperative environments.

As in the perturbation analysis framework response, we additionally considered whether analyzing two categorically distinct classes of environmental compounds—amino acids and simple sugars—could offer insights into the composition of competitive or cooperative environments. This showed that on average there was one more amino acid in competitive environments than in either obligate or facultative cooperative environments in AGORA (14 vs 13), however, this trend did not persist in CarveMe where all environment types considered had the same average of 9 amino acids. In AGORA, each amino acid consistently appeared in a higher proportion of competitive environments than facultative cooperative or obligate, whereas CarveMe did not have this consistent trend. CarveMe also had a higher variation in the proportion of environments that each amino acid appeared in. These results may reflect differences between the collections of metabolic models in the natural habitats where these microbes could be found, but it is also possible that these differences are related to other factors (including model design).

Looking at the simple sugars, we found an average of 4 aldoses/ketoses in all types of environments across both collections. Each individual sugar also appeared in similar proportions of each type of environment. These results indicate that the presence of aldoses/ketoses is not sufficient to distinguish between environments. We now include these analyses in the main text: "*As a case study, we took two groups of environmental compounds that have previously been identified as playing key roles in microbial interactions—amino acids (2,3) and simple sugars (aldoses and ketoses) (37,51)—in Figs. S9 and S10. While we found there was a statistically significant difference in the mean number of amino acids or simple sugars between competitive or cooperative environments, the difference was of the order of 1 compound or less. For example, in CarveMe we would expect to find 9 amino acids in both competitive and cooperative environments (means 9.3 and 8.9, respectively), while in AGORA we found an average of one more amino acid in competitive than cooperative environments (means 14.1 and 13.1, respectively).*" Where, fig. S9 and S10 are additional supplementary figures illustrating the presence of amino acids and simple sugars in competitive and cooperative environments.

> *Integrating environmental similarity metrics with the perturbation framework could enable the authors to test whether resilience and shifts toward cooperation are mediated by compound-level redundancies or larger-scale compositional features. Such an analysis would significantly enhance the biological relevance of the findings.*

Response: The results of our environmental similarity metric analyses indicate that the shifts between cooperation and competition may be related to larger-scale compositional features, but it is not definitive. We are currently exploring, in another project, whether machine learning tools can help us classify these and identify key features. Our initial results suggest that it is still challenging and prediction is by no means perfect. We note though that this work is still quite early in its development and unpublished—indeed it is the current project of a PhD student who is in love with data science.

3. Definitions of Cooperation and Competition

The manuscript defines cooperation as mutual benefit, while competition only requires one organism to experience a negative effect. This asymmetry risks conflating competition with exploitative interactions, where one organism benefits at the expense of another. Exploitative interactions are distinct from pure competition and cooperation, and failing to separate these categories may skew the analysis and interpretation. Providing clear definitions and explicitly distinguishing exploitative interactions would improve the rigor of the study.

Response: We completely agree that there are a range of ecological interactions and it is important to be clear about what we define as cooperation and competition and be aware of possible asymmetries. In terms of metabolic modeling, the results of flux balance analysis give an indication of what might be possible, i.e. the maximum growth rate. Of course what is realized will depend on other factors, such as regulation. So it could be that organisms regulate their metabolism in a "suboptimal" way growing at a rate lower than predicted by metabolic modeling. Within this context, it can be difficult to distinguish between different ecological interactions. For instance, in the case of cooperation we identify scenarios where both organisms could be better off in each other's presence than on their own. However, it is also true that due to regulation or other factors the organisms may not realize this potential. Thus, in our paper we choose to focus on how the environment shapes potential interactions. Cooperative environments are defined as those where there is a potential interaction where both metabolisms grow faster than alone. Competitive environments are defined as those where there is no potential for both metabolisms to grow at the same rate as alone. We have clarified these definitions in the main text, particular in the Introduction.

See: "*Given the many possible sources of competition and cooperation, it can be difficult to resolve the discordance between these studies. For clarity, we focus on environmental resources as an axis along which interactions may vary. In this context, competition arises via limited resources and cooperation arises via the production of new resources or the removal of waste products* " and "*We categorize interactions in terms of their effects on growth by using flux balance analysis to compare the growth rate of metabolisms in pairs and separately in different environments. In this context, cooperation is when both metabolisms grow faster in each other's presence and competition requires that at least one grows slower (i.e. there must be at least one "loser")."*

The reviewer highlights an issue in conflating competition with exploitation. In the metabolic modeling framework there is the potential for exploitation in both what we classify as competitive or cooperative environments. In a competitive environment at least one organism is harmed, but we cannot distinguish between whether both are harmed or only one is harmed. It depends on how resources are allocated and which specific reactions are used, e.g. it could be that one organism can confiscate all of a resource or it could be that the resource is split between both. Metabolic models tend to be plastic/flexible regarding these allocations. In a cooperative environment, both metabolisms can grow faster together than alone, but they do not have to. It might be the case that one metabolism can grow even faster if it harms the other. Indeed if we maximize the sum of the growth rates (a common practice in some metabolic modeling papers), we can find cooperative and exploitative interactions depending on how resources are allocated to metabolisms and what specific reactions are used.

All of this is to say that there are limitations with the capacity of metabolic models to precisely distinguish between ecological interactions. Based on your comments and others, we now highlight these important limitations more explicitly throughout the paper. See for example "*So although we can identify the potential for cooperation or competition, whether these interactions are realized or some other interaction (e.g., exploitation) actually happens will depend on a complex array of factors."* .

It is because of these limitations that we did not choose to identify what precise interaction exists but rather focus on how environments shape the possible interactions. Whether organisms can recognize this potential and how they respond depends on their metabolic strategies, their interactions, and the eco-evolutionary context. This is a very interesting topic—and indeed one we are actively exploring now in follow up work—but beyond the scope of this current study.

4. Beyond Pairwise Interactions

The study focuses exclusively on pairwise interactions, overlooking the complexity of multi-species microbial communities. Multi-species interactions often involve indirect effects and higher-order dynamics that could shift the balance between cooperation and competition. For instance, larger communities might favor cooperation through metabolic complementarity or increase competition due to overlapping resource needs. A discussion of these dynamics, supported by relevant literature, would add critical ecological context to the findings.

Response: Just as microbes do not exist in isolation, nor do they tend to exist with just one partner. Hence, we completely agree that higher order interactions are important to understand the full complexity of multi-species microbial communities. We have now added a paragraph to the Discussion on this topic to add this ecological context to our paper. See "*Furthermore, here we report exclusively on pairwise interactions, but natural microbial communities often contain thousands of interacting species. This could result in higher-order interactions that lead to greater complexity and unpredictability of the interaction dynamics (88). Similar to pairwise dynamics, though, higher-order interactions can result in increased cooperation with new species increasing beneficial interactions within the community (58,89). For example, the addition of a third species has been shown to eliminate the antagonism between a pair of bacteria in a synthetic community of three Bacilota species (90). Alternatively, additional species can result in adverse effects and increased competition (91), such as a switch from a cooperative to an antagonistic two-strain interaction in the presence of a third strain (92). However, some studies show that pairwise co-cultures can partially predict interactions in more complex communities (12,93). Therefore, assessing the types of pairwise species interactions across thousands of environments may provide insights into predicting overall community function.*"

5. Statistical Rigor and Transparency

The manuscript provides limited detail on the statistical framework employed, including whether multiple comparisons were corrected or assumptions were validated. Given the scale of the data, statistical robustness is crucial. The authors should outline their methods in detail, including any corrections, sensitivity analyses, and justifications for sample sizes and thresholds. This transparency will enhance the credibility of the findings.

Response: We have now gone through all the figures in the paper and ensured that they have appropriate statistical tests which are now included in the figure captions:

- In fig. 1B we performed a one-way ANOVA to confirm that the proportions of competitive, cooperative or other interactions did not differ significantly (p-value $> 10\%$) between the 10 bins of 1,000 random pairs in either collection, hence justifying 10,000 random pairs as a representative sample.
- In fig. 2A we performed a one-way ANOVA, as in fig. 1B, to confirm that the proportions of pairs where we could find both competitive and cooperative environments, just competitive, just cooperative or neither did not differ significantly (p-value $> 10\%$) between the 10 bins of 1,000 random pairs in either collection, hence justifying the sample size.
- In fig. 2C we performed a permutation test with 10,000 permutations of the proportions of interactions found in smaller and larger environments to confirm that there is a statistically significant difference between the two environment sizes (p-value < 0.001 in both AGORA and CarveMe).
- In fig. 3 we performed a permutation test, as in fig. 2C, to confirm that there is a statistically significant difference between the proportion of facultative cooperative, one-way obligate, or two-way obligate environments in smaller and larger environments (p-value < 0.001 in both AGORA and CarveMe).
- In fig. 4 we performed an ANOVA with 10 bins to confirm that there was no significant difference between bins in the mean frequency with which the removal of a single compound from the environment would cause a switch to a particular interaction, e.g. competition to facultative cooperation

(p-value $> 10\%$ in both AGORA and CarveMe and both types of starting environment). The apparent mirroring of number of individual compounds that could cause a switch to different types of interactions *vs* those that would cause no change is only a qualitative trend and hence only observed as such (a permutation test with 10,000 permutations shows a significant difference in the frequency of switches, p-value < 0.001).

- In fig. 6 we compared the data against an assumption that each compound in the environment has a fixed role on the interaction between a pair of bacteria. To do this, we used the sensitivity analysis in fig. 4 to set fixed probabilities that removing a single compound from the environment would cause a particular interaction switch. With these fixed probabilities, we ran new simulations on the same number of pairs as in fig. 6 until we observed an interaction switch or all 'substitutable' (not always essential) compounds had been removed from the environment. An ANOVA confirms that the new simulations are statistically significantly different from the data in fig. 6 (p-value < 0.001) showing the impact of environmental compounds on microbial interactions changes as environments degrade. Indeed, this result is also implied by our observation that interactions can switch back and forth between competition and cooperation as compounds are sequentially removed. Our new simulations further show that as environments degrade we predict more obligate interactions than with the fixed probabilities calculated in more diverse environments. This new data has been added as Supp fig. S22.

Minor Points

1. Terminology and Conceptual Clarity
The manuscript occasionally conflates terms such as competition and cooperation with metabolic cross-feeding and resource competition. Since cross-feeding does not always imply cooperation, clear and consistent definitions throughout the text are necessary to avoid confusion.

Response: We have edited our presentation of what we mean competition and cooperation and how we determine them in our metabolic modeling. For example in the Introduction we now state in the second paragraph: "*For clarity, we focus on environmental resources as an axis along which interactions may vary. In this context, competition arises via limited resources and cooperation arises via the production of new resources or the removal of waste products.*" We have also stated what we mean by the term "cross-feeding". It now appears in the first paragraph: "*Yet when two organisms secrete resources needed by the other there can be cooperation, e.g., cross-feeding*". In our modeling framework, cooperation arises via cross-feeding. There can be scenarios, such as commensalism, where one organism benefits but the other is unaffected which we do not classify as cooperation. We understand these terms can mean different things to people from different communities so we have tried to clarify our meaning throughout the text. We now only mention cross-feeding once in the Results and it is in reference to motivating the organisms for our case study.

2. Visualizations
Some figures, particularly the triangular plots (e.g., Figures 2 and 3), are dense and challenging to interpret. Simplifying these visualizations, adding annotations to highlight key trends, and providing more detailed figure legends would improve accessibility.

Response: Based on these suggestions, we have now updated figures 2 and 3. In particular, we have added a schematic to figure 2 to aid the interpretation of the triangle plots and arrows to the axes of all triangles.

We have also updated the figure captions to highlight the key trends. For example, the caption to figure 2 now includes: "*All four plots have the highest density of points in the bottom right corner, shaded in dark green, indicating a high density (≥ 100) where we only found environments for cooperative interactions. A permutation test with 10,000 permutations of the proportions of interactions found in*

smaller and larger environments confirms that there is a statistically significant difference between the two environment sizes (p-value < 0.001 in both AGORA and CarveMe). We found a more diverse spread of interactions in larger environments."

3. B-Vitamin Dependencies

B-vitamin dependencies are mentioned but not fully explored in the discussion. Highlighting the ecological implications of these dependencies, particularly in shaping obligate cooperation in host-associated environments, would strengthen the manuscript.

Response: Thank you for this suggestion. We have now expanded our discussion paragraph on different compounds which commonly caused interaction switches to obligate interactions. It now includes: "*In metabolic models from the CarveMe collection, Fe2+ and Fe3+ ions were major triggers, while in models of gut bacteria from the AGORA collection, many switches to obligacy were due to B vitamin removal. The case of B vitamins is intriguing as in the gut only approximately 12.5% of bacteria are predicted to produce all eight B vitamins (58) and the rest acquire them from the environment or via complementary mechanisms with other bacteria (59,60). Under conditions in which external B vitamins become rare or absent, the survival of these species will crucially depend on their mutually obligate partners or complete B vitamin producers. Thus, our findings linking resource-poor environments with obligate interactions may make sense as those environments may lack compounds that are essential for some bacteria but can be produced via cross-feeding (36,58,61,62)."*

4. Redundancy in Metabolic Models

The models do not appear to account for enzymatic or pathway redundancy, which could buffer microbial interactions against environmental perturbations. Including a brief discussion of how metabolic redundancy influences interaction dynamics would provide additional depth.

Response: We agree with this comment and other suggestions by Reviewer 2 that our Discussion could include further elaboration on the assumptions implicit in our metabolic modeling approach. Consequently, we have added an extra paragraph discussing these themes. In particular, we include: "*Since a common feature in metabolic models is that there are many possible sets of reactions that can give rise to the same growth rate, this plasticity and how it is controlled by regulation will shape the outcome of interactions. This highlights the inherent plasticity of microbial interactions and raises the question of how eco-evolutionary forces determine the interactions that are ultimately realized."*

5. Reproducibility and Data Accessibility

While the manuscript references a GitHub repository for code, it is unclear whether raw data and intermediate analysis outputs are also available. Ensuring full reproducibility by providing these resources is essential.

Response: Thank you for highlighting this. We have now added a sample of metabolic models to the github repository to ensure that our code can be run and methods reproduced. Code for all the statistical analyses of our data is also available here. We have further made this repository permanent and added a license: https://doi.org/10.5281/zenodo.15075550.

6. Compound-Specific Insights

Certain compounds, such as oxygen and nitrate, are identified as key modulators of interaction shifts. Elaborating on the mechanistic basis for these effects would provide additional depth to the discussion.

Response: We have now expanded this discussion point. Previously it read: "*The availability of terminal electron acceptors (e.g., oxygen, nitrate, and nitrite) also played a major role in causing switches, reflecting diversity in different bacteria's ability to use anaerobic respiration and fermentation products.* " And now: "*The removal of terminal electron acceptors (e.g., oxygen, nitrate, and nitrite) also played a major role, as it forces bacteria to use alternative metabolic pathways with, e.g., anaerobic respiration and fermentation products (68).* "

> *7. Language and Style*
> *The manuscript occasionally uses jargon-heavy language that could deter readers. Simplifying such phrases without losing scientific rigor would improve readability.*

Response: We have gone through the manuscript and tried to remove jargon. One example is that in the first paragraph of the introduction we removed the phrase "syntrophic networks". We also reworked the description of the gut microbiota paragraph to make it more general and improve the flow. It now reads: "*Similarly, in the human gut dietary changes can have significant impacts on communities of microbes. Normally a variety of dietary fibers maintains a complex and diverse microbial community, but restricted fiber consumption can lead to the decline of fiber-restricted specialists in place of generalists, that then switch to the mucus barrier of the host as an alternative energy source.* "

Reviewer 2

> *In their manuscript "Competition and cooperation: the plasticity of bacterial interactions across environments", Solowiej-Wedderburn J et al. use two metabolic network databases to investigate the effect of growing pairs of microbes together. Such databases only contain the metabolic information of individual microbes. Thus, Solowiej-Wedderburn J et al. theoretical approach relies on joining those individual metabolisms and measuring the effect on their maximum individual growth rate (hereafter interaction). By doing so, they are faced with the question of which extracellular compounds (hereafter environment) are essential to sustaining the pair's life. They test a large and random set of compounds, finding that growth rates can be affected in all sorts of ways, including cooperation (+/+), competition (-/-) and others, including neutral (=/=). Their high-throughput approach is sound and addresses an important question with implications for microbial ecology and evolution. I enjoyed reading this work, which would be a valuable contribution to PLoS Computational Biology. However, some model assumptions and implications seem to require further elaboration. Find a point-by-point list below.*

Response: Thank you for the positive response to the theoretical approach and results of our analyses. We have made revisions to address issues concerning the assumptions and implications and discuss them in subsequent responses.

Major Points

> *1. Defining cooperation and competition is essential. However, that these are linked to the growth rate is clarified too late. I suggest defining them from the introduction and talking about the growth rate earlier (abstract and introduction), as readers expect to find this information early on. Actually, referring to growth rate seems more natural than referring to cooperation and competition (more ambiguous terms) sometimes. Also, you could mention the pros/cons of using growth rate to define cooperation/competition and whether your results are robust to this choice.*

Response: Thank you for this suggestion. Based on this comment and similar ones by the editor and other referee, we have revised our Introduction to bring the definitions of competition and cooperation forward in our paper. We also highlight that we focus on growth rates as a metric for classifying interactions. Here is an excerpt from the last paragraph of the Introduction where we lay out the framework used in the paper. "*We categorize interactions in terms of their effects on growth by using flux balance analysis to compare the growth rate of metabolisms in pairs and separately in different environments. In this context, cooperation is when both metabolisms grow faster in each other's presence and competition requires that at least one grows slower (i.e. there must be at least one "loser"). We vary the composition and the number of compounds in environments to elucidate general principles concerning how environments affect the likelihood of competition and cooperation.* "

> *2. Your approach scans an impressive number of environments and microbial pairs. However, early on, one wonders about the assumptions made and their implications when comparing those different environments and microbial pairs. For example, you could mention that temperature, pH, and gene/expression regulation are not considered.*

Response: Yes, that is a good point. We have now added some clarification in the Introduction to specify exactly what we are considering. It now reads "*Adding to the complexity, studies have indicated that environmental factors such as temperature, toxicity, and resource availability can all contribute to the interactions between pairs of microbes and that sometimes the same microbes can exhibit vastly different types of interactions under different conditions (18– 21). Given the many possible sources of competition and cooperation, it can be difficult to resolve the discordance between these studies. For clarity, we focus on environmental resources as an axis along which interactions may vary.*"

> *3. It would be helpful to make it more clear whether your model is dynamic or if it assumes an equilibrium. This is particularly important for the section where you degrade the environment one compound at a time. Currently, many of the model assumptions and critical details are restricted to the methods section, but they are important for interpreting results and discussion at times.*

Response: Yes, flux balance analysis assumes that metabolisms (or populations) are in growth phase. They are not dynamic— at least in the sense that the metabolisms and the environmental composition are not changing. Based on the results of flux balance analysis, one could create a dynamic or evolutionary model but we did not do that here. We make these points in a new paragraph in the Discussion. See for example: "*It [flux balance analysis] also assumes that species (or metabolisms) are static and not evolving in ways that would change their metabolism. While metabolic models could be incorporated into an eco-evolutionary, dynamic framework , we do not do so here.*"

> *4. Like other metabolic models, you assume the growth rate is maximized. This assumption should be stated more visibly. In fact, you could mention more model limitations, assumptions or biases in your discussion.*

Response: Thank you for this suggestion! We now include a new paragraph discussing these assumptions more explicitly. It starts with: "*We also note that our use of flux balance analysis to infer metabolic interactions entails certain assumptions. For one, it focuses on those competitive and cooperative interactions that alter growth rate, specifically in actively growing populations. It also assumes that species (or metabolisms) are static and not evolving in ways that would change their metabolism.. . .* "

> *5. The magnitude of your work offers an excellent opportunity for an ample discussion about the eco-evolutionary implications of cooperation and competition. This would be very satisfying to see for many readers.*

Response: Thanks. We mention these possibilities now in a new paragraph in the Discussion. For example, see: "*This highlights the inherent plasticity of microbial interactions and raises the question of how eco-evolutionary forces determine the interactions that are ultimately realized.*"

Minor Points

6. In your abstract, please make it clear that this is a theoretical work.

Response: We have accordingly amended the abstract: "*By computationally simulating thousands of environments for 10,000 pairs of bacteria from each collection, we found that most pairs were able to both compete and cooperate depending on the availability of environmental resources. This modeling approach allowed us to determine commonalities between environments that could facilitate the potential for cooperation or competition between a pair of species.* "

7. You could briefly mention alternative explanations for the plasticity of interactions apart from the environment.

Response: One possible source of plasticity is in the metabolic models themselves. There are many possible ways to realize the same growth rate, and which specific reactions are used will determine what interactions are realized. Thus, there is the plasticity in what interactions are possible across environments and there is plasticity in what interactions are possible in a particular environment.

We now include mention of plasticity in a new paragraph in the Discussion. See "*Our results reveal that in the majority of cases the same pair of bacteria can exhibit either cooperation or competition, depending on relatively small changes in the resources present in an environment. Importantly, our metabolic modeling only demonstrates what interactions are possible— what actually occurs will depend on which specific reactions are used. Since a common feature in metabolic models is that there are many possible sets of reactions that can give rise to the same growth rate, this plasticity and how it is controlled by regulation will shape the outcome of interactions. This highlights the inherent plasticity of microbial interactions and raises the question of how eco-evolutionary forces determine the interactions that are ultimately realized.*"

8. Your work cites previous work on metabolic modelling. I would mention the distinction between your work and that literature more clearly: stating your contribution more plainly.

Response: Thank you for this suggestion. We have now stated our contribution more plainly, adding these sentences to the introduction where we discuss the literature: "*However, metabolic modeling has typically been used to examine specific sets of organisms or specific environmental contexts. By harnessing the computational tractability of these metabolic models and leveraging the huge number of models now available, we can interrogate the role the environment plays in shaping competition and cooperation more broadly, on a scale that would be difficult to evaluate empirically.*"

9. In the results, one wonders if neutral interactions have to have the exact same growth rate or if you have a threshold of change to call them neutral. Please clarify this.

Response: Yes, we include a threshold of ± 0.001 to define neutral interactions. This is now included in the caption of table 1 where interactions are defined.

10. For most of the introduction and results, you do not mention what the "compounds" are. To make this less abstract, saying that these compounds are proteins, sugars, fatty acids, vitamins, etc, would be useful. Also, how complex are these compounds? How different are they between the microbes in a pair?

Response: We have now offered a few examples of environmental compounds in the first paragraph of the Results: "*By modifying the presence and abundance of these compounds (e.g., amino acids, carbohydrates, or metal ions).* " We have additionally provided some specific data on the percentage of competitive and cooperative environments that two specific groups of compounds—amino acids and simple sugars (aldoses and ketoses)—appear in (Supp figs. S9 and S10) and, further, their significance in interaction switches (Supp figs. S13 and S14) to remove some of this abstraction. We uploaded the full set of environmental compounds to the github repository for this paper which can be found here: https://doi.org/10.5281/zenodo.15075550.

11. Although you talk about the environment, that interactions switch based only on non-essential compounds is not very clearly stated. This could be mentioned more explicitly, as this is quite interesting.

Response: Thank you for this suggestion. We have now stated this more explicitly in the Results section when describing the framework for the environmental degradation analysis: "*Furthermore, we never removed the compounds that had previously been identified as essential across all environments for that pair.*" We have also included an additional schematic in the supplement (fig. S1) to clarify our classification of compounds as essential or substitutable, and used or redundant.

12. When you investigate the switch between cooperation and competition, it would be helpful to try to explain one of these occurrences mechanistically. Also, it would be interesting to know if the mechanism is the same between different microbial pairs for one of these compounds leading to a switch.

Response: We think this is an excellent idea. In order to do this for any individual switch event, we could identify the metabolic reactions in both microbes that involve the compound that caused the switch. Further, running the flux balance analysis would give a set of reactions that are being used by the microbes before and after the compound is removed (however, it is important to note that while the maximized growth rate has a unique solution, the reaction fluxes to achieve this are not unique). Ideally, these analyses would identify consistent links between particular compounds, the pathways they are used in, and consequence on microbial interactions. For example, if two microbes have optimal pathways that both rely on the same compound, they would compete for it; on the removal of that compound the microbes would be forced to choose different pathways which might open up opportunities for cooperation if the alternative pathways produce favorable by-products. In the case of a switch to obligate interactions, one possible scenario is that the given compound is critical for the growth of one of the microbes which is unable to synthesize it itself (is auxotrophic). Hence, if the compound is removed the microbe becomes obligate on its partner (who in this scenario is able to synthesize and export the compound).

We investigated the case studies in Fig. 5 in search of such mechanisms. We focused on switches to obligate interactions as we thought that these would present the cleanest story. Among these examples we could not identify any immediate auxotrophies as outlined above. However, we still found many examples of obligate interactions in our case studies. For example, of the 70 cases of switches to obligate interactions between *Prevotella copri DSM 18205* and *Ruminococcus gnavus AGR2154*, 30 were caused by the removal of L-glutamine and 13 by the removal of L-glutamate(1-)—two amino acids which are present in the biomass reactions of both bacteria indicating that they are necessary for growth. While the *Ruminococcus* strain can import/export both L-glutamine and L-glutamate, the *Prevotella* strain can only import/export L-glutamate. Both can convert L-glutamine to L-glutamate and vice versa. We found that in some environments lacking both L-glutamate and L-glutamine, *Prevotella* was still able to synthesize both amino acids from the other environmental compounds present; however, it was often the case that while *Ruminococcus* could make up for a lack of either L-glutamine or L-glutamate in the environment it could not grow without both. In such cases, when L-glutamine was removed, *Prevotella* which lacks a transport reaction for L-glutamine continued to be able to grow, whereas *Ruminococcus* could no longer grow by itself but became obligate on *Prevotella*.

Delving deeper into the mechanisms, we sought to identify which metabolic reactions both species used in the 30 cases when *Ruminococcus* became obligate on *Prevotella* when L-glutamine was removed. Each of these environments corresponded to the same initial environment lacking in L-glutamate but with different sequences of compounds removed (fig. 5G). Here, we observed an average of 16.7 reactions (range 8–26, spanning 81 unique reactions) that *Prevotella* began to use after L-glutamine was removed from the environment, while *Ruminococcus* stopped using an average of 10.2 reactions (range 6–16, spanning 80 unique reactions) and started using an average of 11.1 reactions (range 5–19, spanning 85 unique reactions) after L-glutamine was removed. In some cases, it is clear to see how the obligate relation could be realized, for example, in 11/30 cases *Prevotella* began to export L-glutamate after L-glutamine was removed which could then directly be imported and used by *Ruminococcus*. However, even in these cases, providing a full explanation of all the internal metabolic reactions that start/stop being used with/without L-glutamine is less obvious. Indeed, to explain each reaction change may not be entirely meaningful since the flux balance analysis only outputs one possible set of reactions that could give the maximized growth output, whereas there may be many alternative metabolic reactions or pathways that give the same result. For some specific cases of lab-cultured strains metabolic models have been carefully curated to partition reactions into different pathways and incorporate regulatory mechanisms (e.g. https://doi.org/10.1038/msb.2011.65, https://doi.org/10.1186/1752-0509-3-38, https://doi.org/10.1371/journal.pcbi.1001002), but this has not been done on a broader scale. As metabolic models become refined, we can hope to achieve stronger predictions and better understandings of the complex interplay between resource availability, metabolic pathways and their effect on microbial interactions.

Our analysis further highlighted the environmental context of interaction switches. For example, we found 5 cases where the removal of deoxycytidine resulted in a switch competition/cooperation to an obligate interaction between *Prevotella Ruminococcus*, however, in 10 cases it could be removed with no effect (here, competitive interactions remained competitive). Furthermore, when deoxycytidine was removed from environments with *Prevotella* and *Bacteroides vulgatus ATCC 8482* there was no change in the majority of cases—31 competitive interactions remained competitive and 29 cooperative interactions remained cooperative—however, we also found 1 case where the removal of deoxycytidine could switch their interaction from facultative cooperation to competition and 15 cases where its removal switched the interaction to obligate. In the context of these case studies, environments only differ by a few compounds and yet we can see a variety of interaction shifts. Furthermore, in many cases, we observed changes in over 200 reactions of a single metabolism when a single compound was removed, hence making it difficult to isolate a single critical metabolic pathway responsible for an interaction switch. In the same way that we found a range of different metabolic mechanisms that could account for interaction switches between a specific pair, there was an even wider range of possible mechanisms between different pairings.

In light of this and other comments by the reviewers (e.g. reviewer 2's major points 4 and 5 on model limitations and eco-evolutionary implications) we have amended the discussion to highlight some of this complexity. See, e.g., "*Since a common feature in metabolic models is that there are many possible sets of reactions that can give rise to the same growth rate, this plasticity and how it is controlled by regulation will shape the outcome of interactions.*"

For the succinctness of the paper we chose to focus on the general pattern that the most common compounds causing switches are key metabolites with well established roles in governing microbial interactions in the main text. However, we thought that this discussion could prove interesting and add value to the reader so have included it as supplementary text A in the Supporting Information.

13. The explanation after Equation (2) regarding the growth inequality is a little convoluted. Please simplify/clarify it.

Response: We have now clarified the explanation of the growth inequality in equation 2 to: "*The linear program Eq.(2) for a pair includes an additional constraint to ensure that while optimizing the growth rate of individual M_i, the growth rate of the second species ($\lambda^{\dagger}_{M_j}$) is no worse than when it was alone (λ_{M_j}, determined by solving Eq.(1) for M_j).*"

14. In Fig. 5, the caption mentions ”neutral interactions in blue”, but that is not shown.

Response: We have now removed the text “neutral interactions in blue” from the figure caption.

15. In Fig 4, you could break the y-axis into two sections: 1) from 0 to 30 to show obl, f comp, other and no grow, and 2) from 70 to 100 to show stay. This would enable seeing the differences in the range 0 to 30 better.

Response: We agree that fig. 4 could be presented more clearly to allow the reader to both appreciate that most compounds, if individually removed, would cause no change to the interaction between a pair while highlighting that on average there is at least one compound that could be removed from an environment causing a switch. As such we have updated the figure:

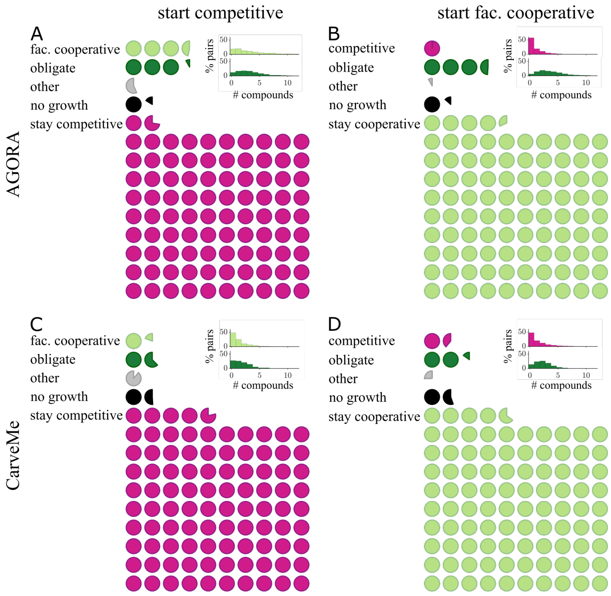

Figure 4: **Stability of interactions to the loss of a single compound.** A–D) Circles summarize the average effect on the interaction between a pair of bacteria when a single compound is removed from the environment. In (A) and (C) the initial environment is competitive, and in (B) and (D) facultative cooperative. Specifically, 1,000 pairs of bacteria in AGORA (A and B) or CarveMe (C and D) are considered; for each pair we select one competitive and one facultative cooperative initial environment with 100 additional compounds, determine the effect when each of these compounds is individually removed from the starting environment (see *Methods: Single removal of compounds*). The circles show the the mean frequency that removing a single compound from the environment would result in a switch to a different type of interaction or stay the same (separating the data into 10 bins confirms there is no significant difference between bins, p-value $> 10\%$). In the majority of cases, if a single compound is removed from the environment the interaction between the pair of bacteria would remain the same. However, on average there is at least one compound which would result in a switch from a competitive to a cooperative environment or vice versa. Qualitatively, the results for initial facultative cooperative environments appear to mirror the results when starting in competitive environments for both collections. Inserted histograms show that for 70-83% pairs there are 1–6 compounds which can cause a switch to obligate interactions; while for 41–60% pairs there are 1–6 compounds which can cause an interaction switch between competition and facultative cooperation if individually removed.

16. I find it interesting that you could separate the effect of the environment and microbial partner on the growth rate. For example, measuring the growth of microbe i when 1) all

compounds needed by microbe j are added without adding microbe j itself, and discount this from the case where 2) all compounds needed by microbe j and microbe j itself are added.

Response: Yes, this is exactly what we did as a way of assessing the type of interaction. We now make this clearer in the Introduction. "*We categorize interactions in terms of their effects on growth by using flux balance analysis to compare the growth rate of metabolisms in pairs and separately in different environments.*"

17. Please deposit your code and data in a permanent repository (such as Zenodo) and consider adding a license. See, for example, https://docs.github.com/en/repositories/archiving-a-github-repository/referencing-and-citing-content and https://docs.github.com/en/communities/setting-up-your-project-for-healthy-contributions/adding-a-license-to-a-repository

Response: Thank you for making these suggestions and directing us to Zenodo. We have now followed your recommendation and our code and sample of metabolic models can be found here: https://doi.org/10.5281/zenodo.15075550.
